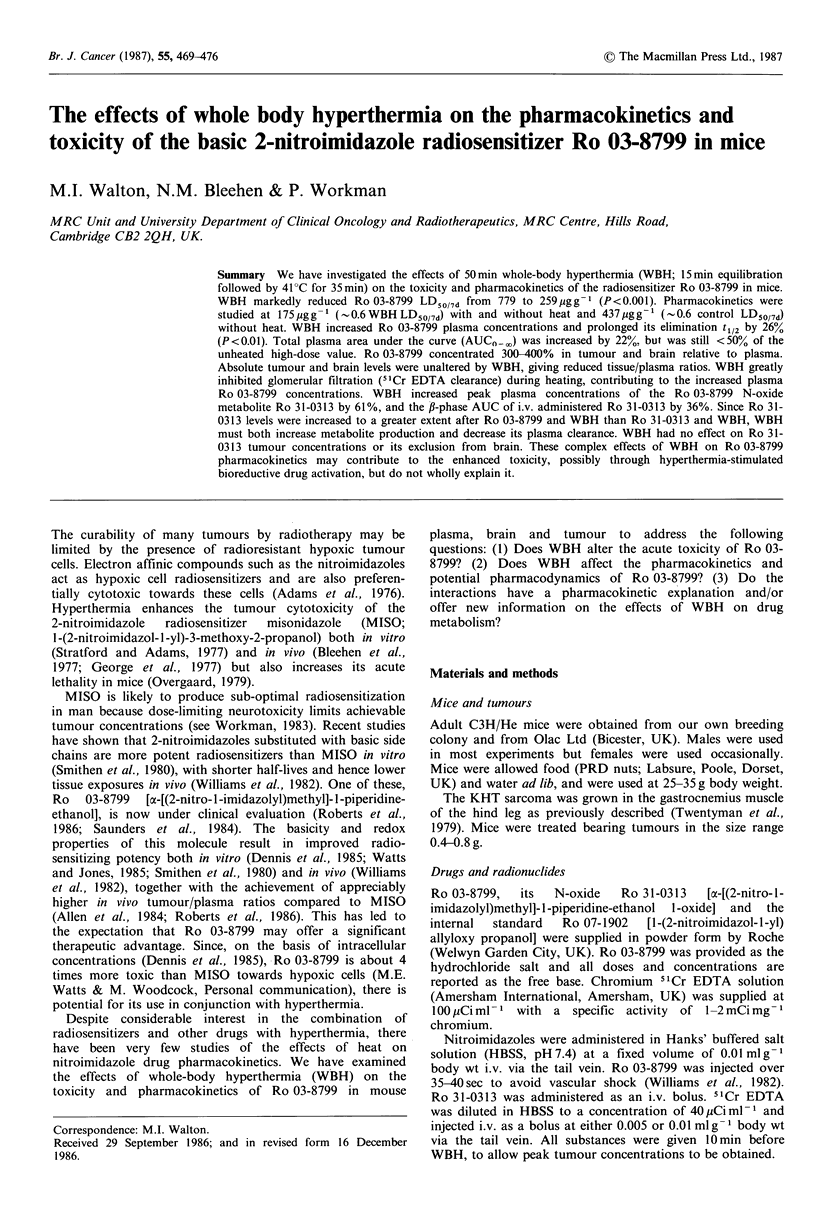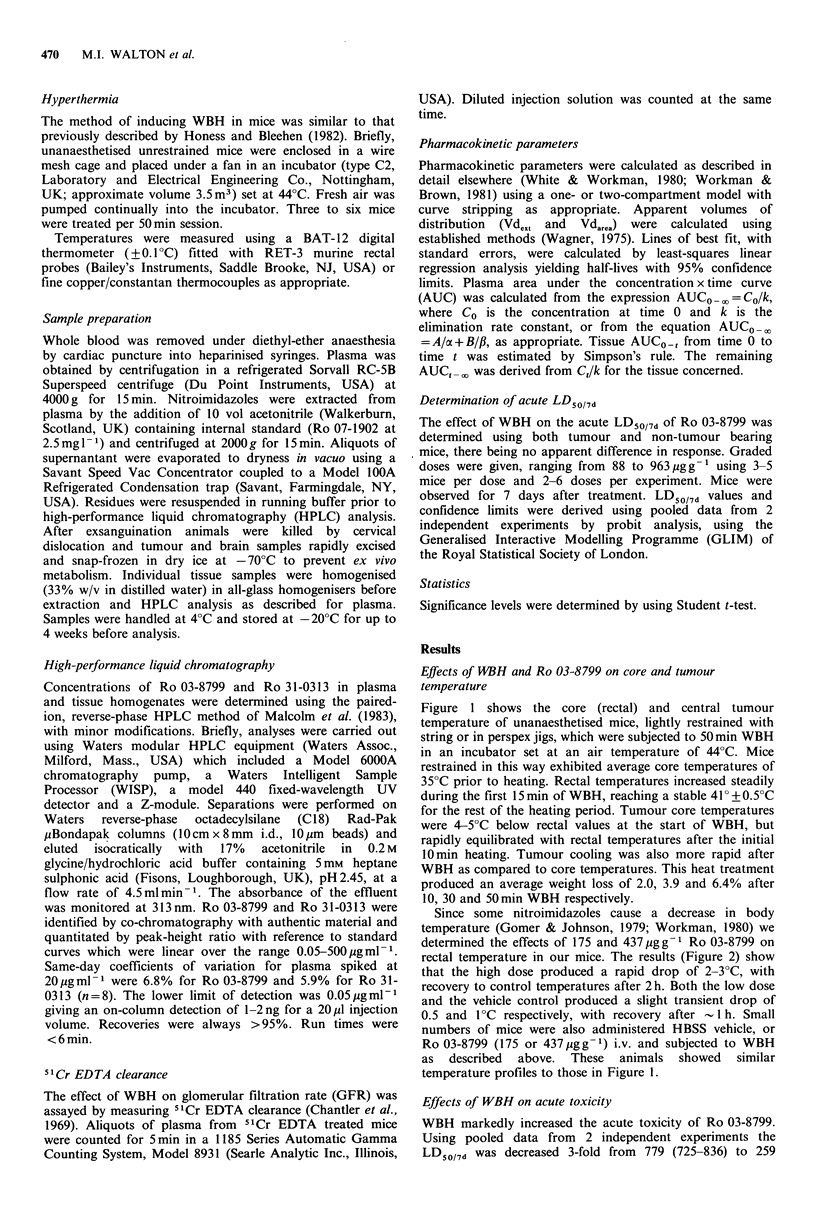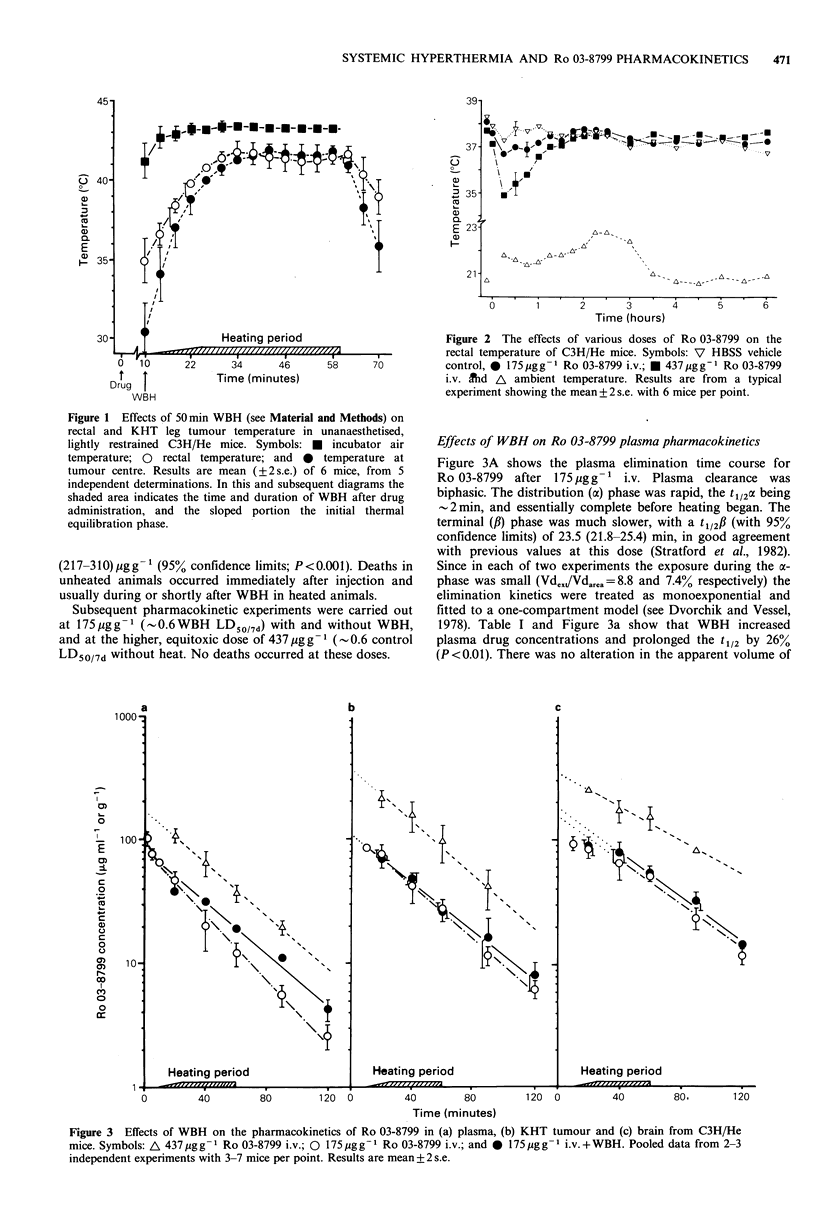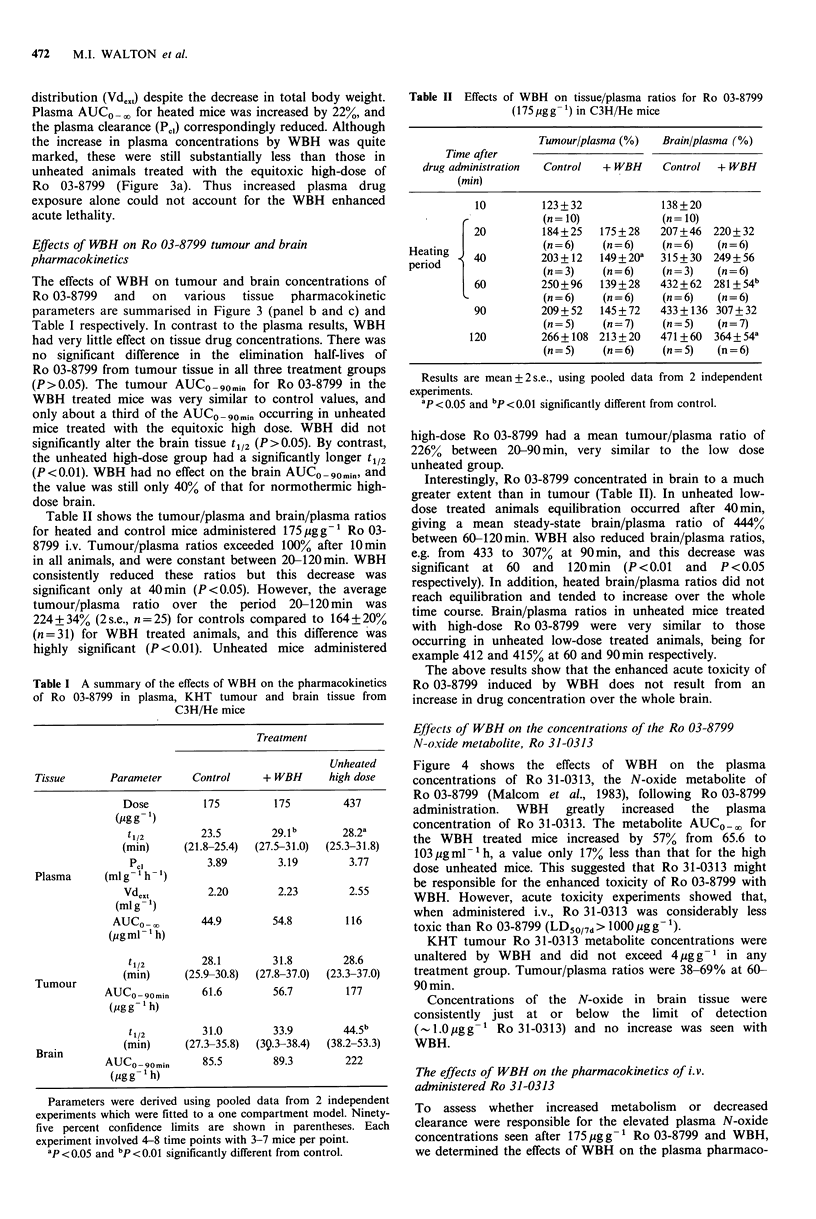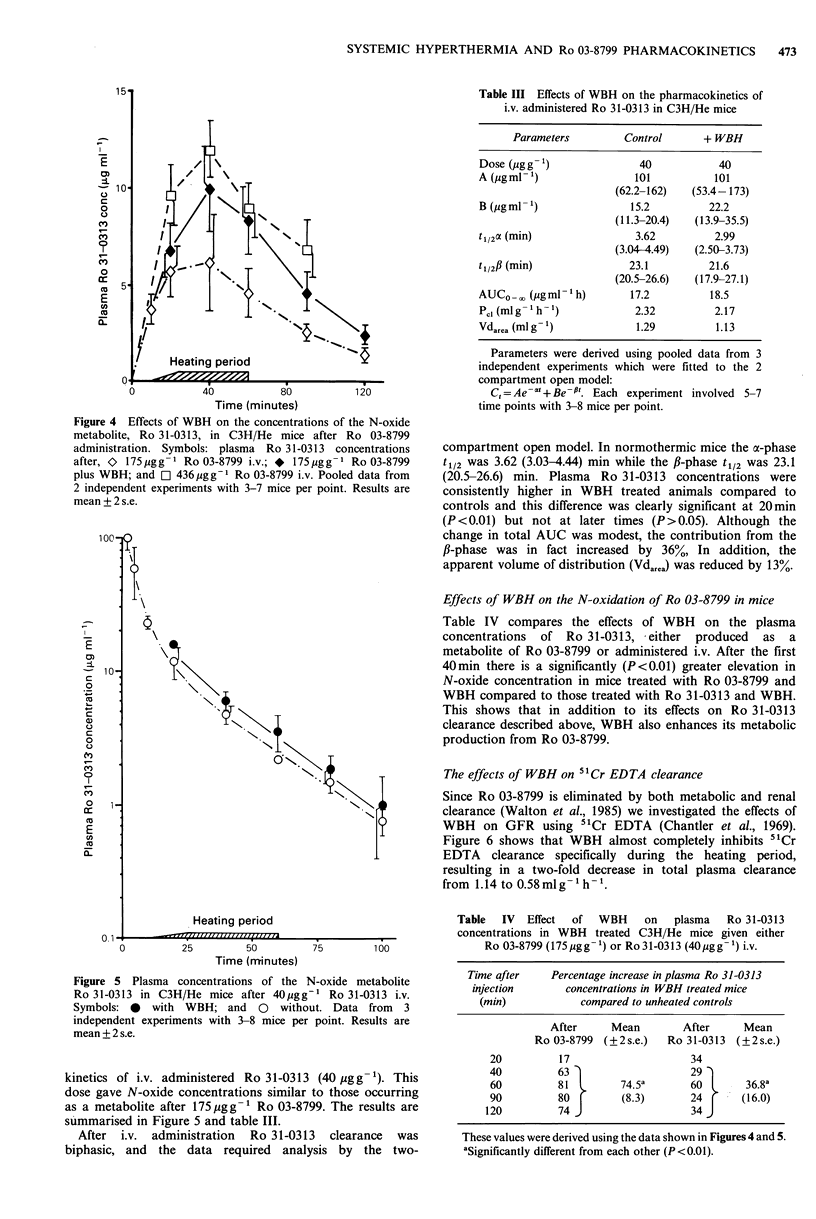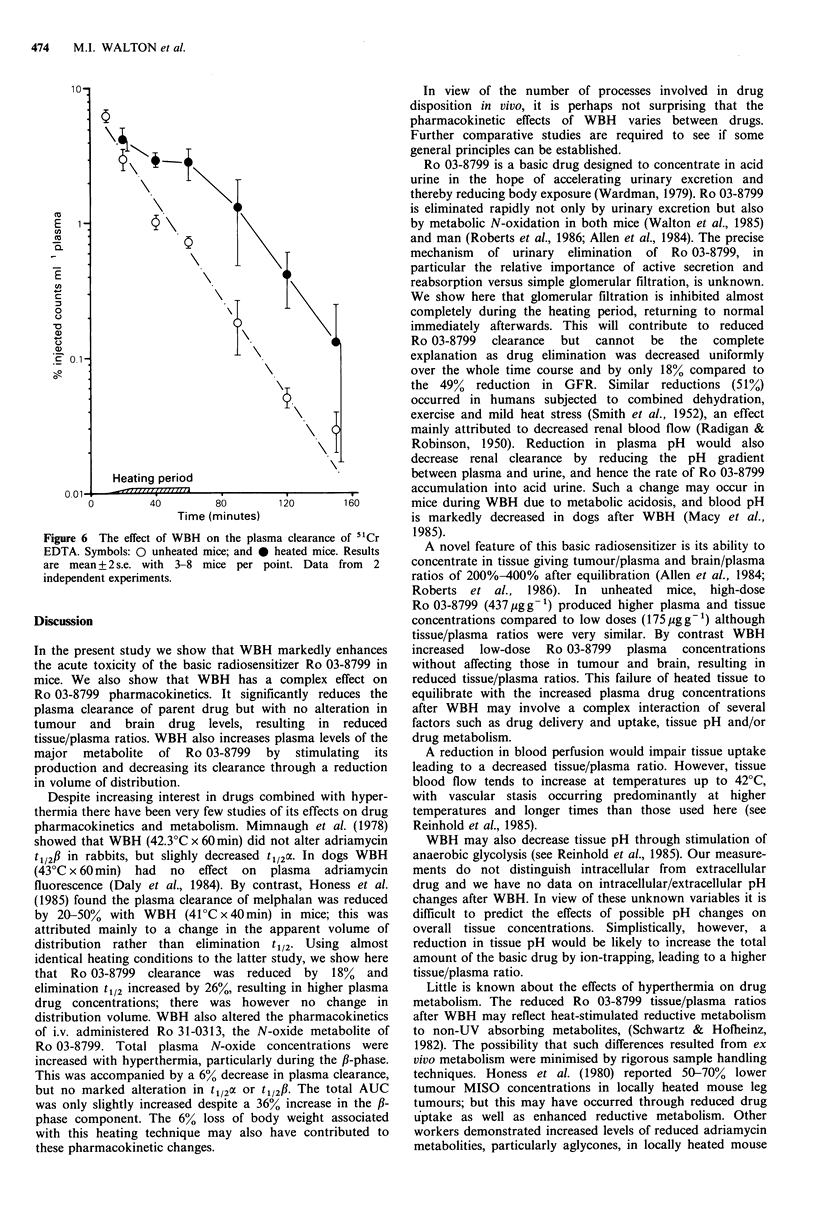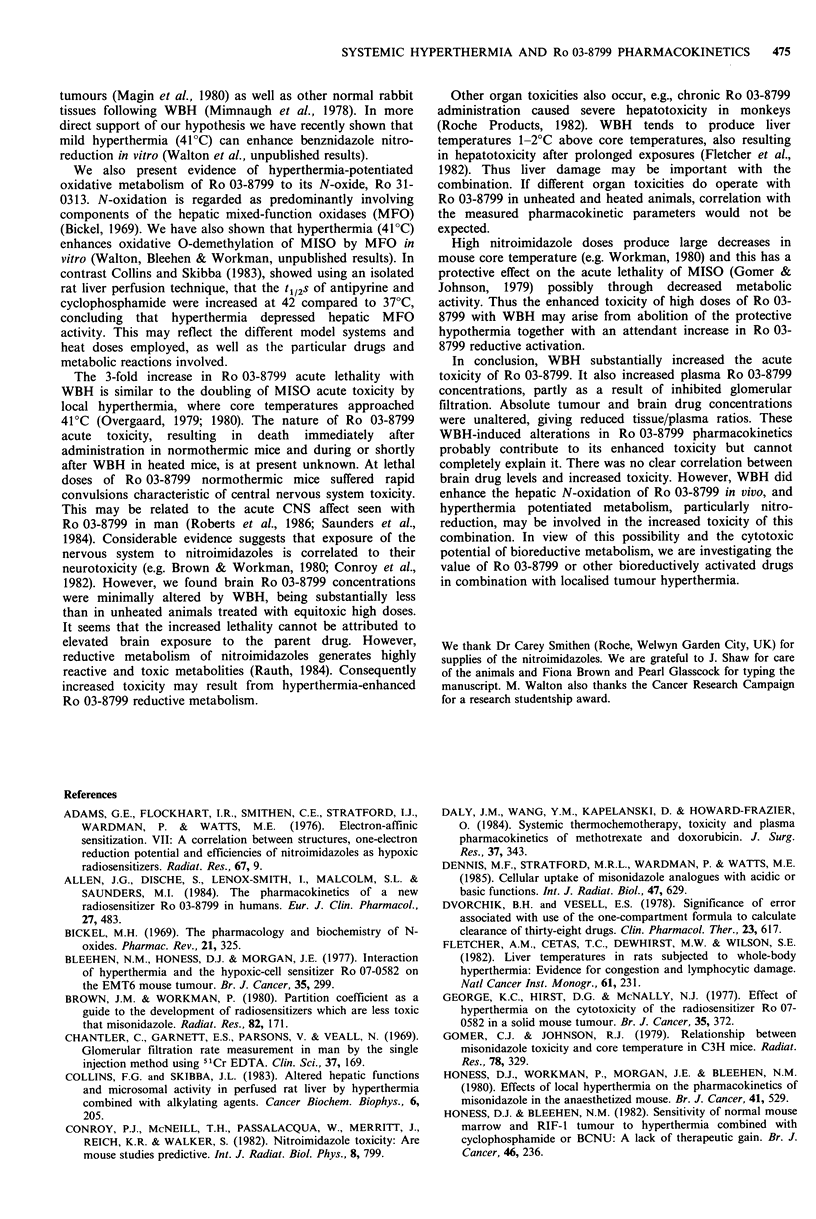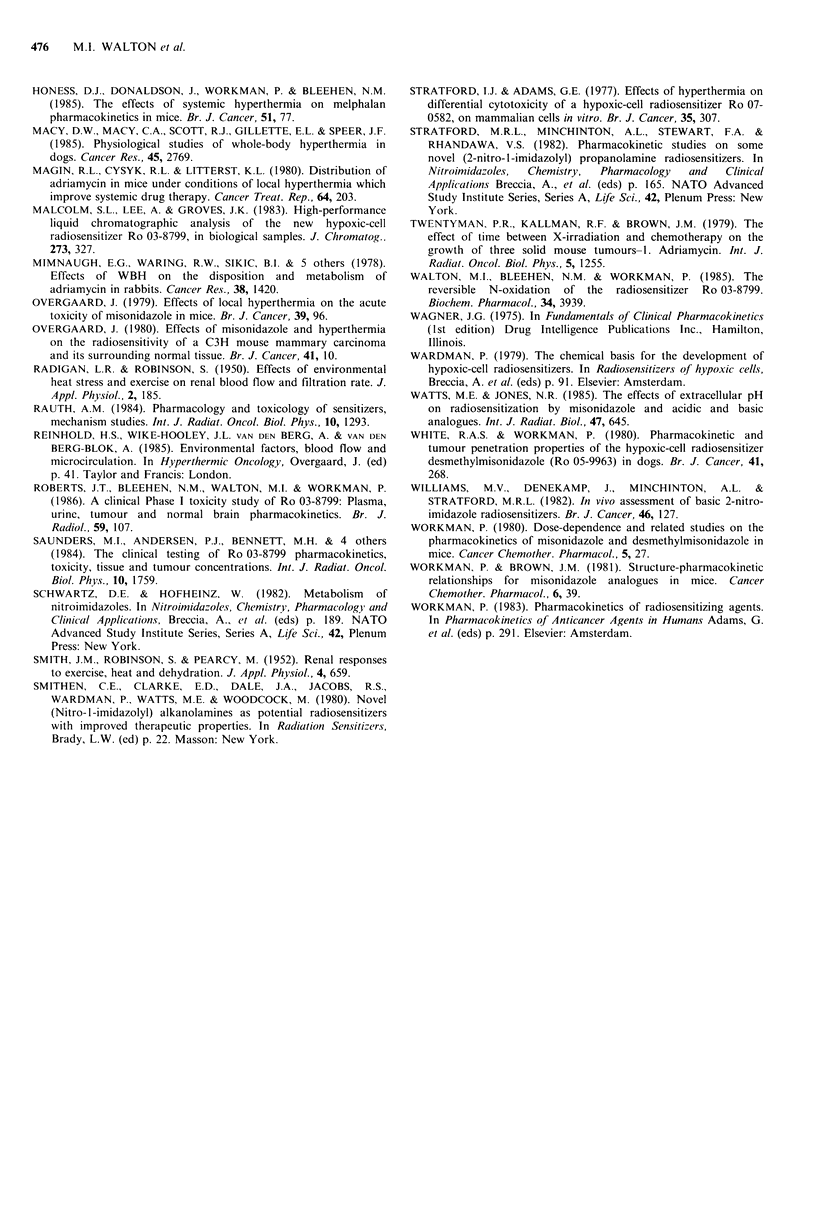# The effects of whole body hyperthermia on the pharmacokinetics and toxicity of the basic 2-nitroimidazole radiosensitizer Ro 03-8799 in mice.

**DOI:** 10.1038/bjc.1987.96

**Published:** 1987-05

**Authors:** M. I. Walton, N. M. Bleehen, P. Workman

## Abstract

We have investigated the effects of 50 min whole-body hyperthermia (WBH; 15 min equilibration followed by 41 degrees C for 35 min) on the toxicity and pharmacokinetics of the radiosensitizer Ro 03-8799 in mice. WBH markedly reduced Ro 03-8799 LD50/7d from 779 to 259 micrograms g-1 (P less than 0.001). Pharmacokinetics were studied at 175 micrograms g-1 (approximately 0.6 WBH LD50/7d) with and without heat and 437 micrograms g-1 (approximately 0.6 control LD50/7d) without heat. WBH increased Ro 03-8799 plasma concentrations and prolonged its elimination t1/2 by 26% (P less than 0.01). Total plasma area under the curve (AUC0-infinity) was increased by 22%, but was still less than 50% of the unheated high-dose value. Ro 03-8799 concentrated 300-400% in tumour and brain relative to plasma. Absolute tumour and brain levels were unaltered by WBH, giving reduced tissue/plasma ratios. WBH greatly inhibited glomerular filtration (51Cr EDTA clearance) during heating, contributing to the increased plasma Ro 03-8799 concentrations. WBH increased peak plasma concentrations of the Ro 03-8799 N-oxide metabolite Ro 31-0313 by 61% and the beta-phase AUC of i.v. administered Ro 31-0313 by 36%. Since Ro 31-0313 levels were increased to a greater extent after Ro 03-8799 and WBH than Ro 31-0313 and WBH, WBH must both increase metabolite production and decrease its plasma clearance. WBH had no effect on Ro 31-0313 tumour concentrations or its exclusion from brain. These complex effects of WBH on Ro 03-8799 pharmacokinetics may contribute to the enhanced toxicity, possibly through hyperthermia-stimulated bioreductive drug activation, but do not wholly explain it.


*Br. J. Cancer* (1987), **55**, 469–476 © The Macmillan Press Ltd., 1987

### The effects of whole body hyperthermia on the pharmacokinetics and toxicity of the basic 2-nitroimidazole radiosensitizer Ro 03-8799 in mice

M.I. Walton, N.M. Bleehen & P. Workman

*MRC Unit and University Department of Clinical Oncology and Radiotherapeutics, MRC Centre, Hills Road, Cambridge CB2 2QH, UK.*

**Summary** We have investigated the effects of 50 min whole-body hyperthermia (WBH; 15 min equilibration followed by 41°C for 35 min) on the toxicity and pharmacokinetics of the radiosensitizer Ro 03-8799 in mice. WBH markedly reduced Ro 03-8799 $LD_{50/7d}$ from 779 to 259 $\mu g g^{-1}$ ($P<0.001$). Pharmacokinetics were studied at 175 $\mu g g^{-1}$ (~0.6 WBH $LD_{50/7d}$) with and without heat and 437 $\mu g g^{-1}$ (~0.6 control $LD_{50/7d}$) without heat. WBH increased Ro 03-8799 plasma concentrations and prolonged its elimination $t_{1/2}$ by 26% ($P<0.01$). Total plasma area under the curve ($AUC_{0-\infty}$) was increased by 22%, but was still <50% of the unheated high-dose value. Ro 03-8799 concentrated 300–400% in tumour and brain relative to plasma. Absolute tumour and brain levels were unaltered by WBH, giving reduced tissue/plasma ratios. WBH greatly inhibited glomerular filtration ($^{51}Cr$ EDTA clearance) during heating, contributing to the increased plasma Ro 03-8799 concentrations. WBH increased peak plasma concentrations of the Ro 03-8799 N-oxide metabolite Ro 31-0313 by 61%, and the $\beta$-phase AUC of i.v. administered Ro 31-0313 by 36%. Since Ro 31-0313 levels were increased to a greater extent after Ro 03-8799 and WBH than Ro 31-0313 and WBH, WBH must both increase metabolite production and decrease its plasma clearance. WBH had no effect on Ro 31-0313 tumour concentrations or its exclusion from brain. These complex effects of WBH on Ro 03-8799 pharmacokinetics may contribute to the enhanced toxicity, possibly through hyperthermia-stimulated bioreductive drug activation, but do not wholly explain it.

The curability of many tumours by radiotherapy may be limited by the presence of radioresistant hypoxic tumour cells. Electron affinic compounds such as the nitroimidazoles act as hypoxic cell radiosensitizers and are also preferentially cytotoxic towards these cells (Adams *et al.*, 1976). Hyperthermia enhances the tumour cytotoxicity of the 2-nitroimidazole radiosensitizer misonidazole (MISO; 1-(2-nitroimidazol-1-yl)-3-methoxy-2-propanol) both *in vitro* (Stratford and Adams, 1977) and *in vivo* (Bleehen *et al.*, 1977; George *et al.*, 1977) but also increases its acute lethality in mice (Overgaard, 1979).

MISO is likely to produce sub-optimal radiosensitization in man because dose-limiting neurotoxicity limits achievable tumour concentrations (see Workman, 1983). Recent studies have shown that 2-nitroimidazoles substituted with basic side chains are more potent radiosensitizers than MISO *in vitro* (Smithen *et al.*, 1980), with shorter half-lives and hence lower tissue exposures *in vivo* (Williams *et al.*, 1982). One of these, Ro 03-8799 [α-[(2-nitro-1-imidazolyl)methyl]-1-piperidine-ethanol], is now under clinical evaluation (Roberts *et al.*, 1986; Saunders *et al.*, 1984). The basicity and redox properties of this molecule result in improved radiosensitizing potency both *in vitro* (Dennis *et al.*, 1985; Watts and Jones, 1985; Smithen *et al.*, 1980) and *in vivo* (Williams *et al.*, 1982), together with the achievement of appreciably higher *in vivo* tumour/plasma ratios compared to MISO (Allen *et al.*, 1984; Roberts *et al.*, 1986). This has led to the expectation that Ro 03-8799 may offer a significant therapeutic advantage. Since, on the basis of intracellular concentrations (Dennis *et al.*, 1985), Ro 03-8799 is about 4 times more toxic than MISO towards hypoxic cells (M.E. Watts & M. Woodcock, Personal communication), there is potential for its use in conjunction with hyperthermia.

Despite considerable interest in the combination of radiosensitizers and other drugs with hyperthermia, there have been very few studies of the effects of heat on nitroimidazole drug pharmacokinetics. We have examined the effects of whole-body hyperthermia (WBH) on the toxicity and pharmacokinetics of Ro 03-8799 in mouse plasma, brain and tumour to address the following questions: (1) Does WBH alter the acute toxicity of Ro 03-8799? (2) Does WBH affect the pharmacokinetics and potential pharmacodynamics of Ro 03-8799? (3) Do the interactions have a pharmacokinetic explanation and/or offer new information on the effects of WBH on drug metabolism?

Correspondence: M.I. Walton.
Received 29 September 1986; and in revised form 16 December 1986.

### Materials and methods

### *Mice and tumours*

Adult C3H/He mice were obtained from our own breeding colony and from Olac Ltd (Bicester, UK). Males were used in most experiments but females were used occasionally. Mice were allowed food (PRD nuts; Labsure, Poole, Dorset, UK) and water *ad lib*, and were used at 25–35 g body weight.

The KHT sarcoma was grown in the gastrocnemius muscle of the hind leg as previously described (Twentyman *et al.*, 1979). Mice were treated bearing tumours in the size range 0.4–0.8 g.

### *Drugs and radionuclides*

Ro 03-8799, its N-oxide Ro 31-0313 [α-[(2-nitro-1-imidazolyl)methyl]-1-piperidine-ethanol 1-oxide] and the internal standard Ro 07-1902 [1-(2-nitroimidazol-1-yl) allyloxy propanol] were supplied in powder form by Roche (Welwyn Garden City, UK). Ro 03-8799 was provided as the hydrochloride salt and all doses and concentrations are reported as the free base. Chromium $^{51}Cr$ EDTA solution (Amersham International, Amersham, UK) was supplied at 100 $\mu Ci\,ml^{-1}$ with a specific activity of 1–2 $mCi\,mg^{-1}$ chromium.

Nitroimidazoles were administered in Hanks' buffered salt solution (HBSS, pH 7.4) at a fixed volume of 0.01 $ml\,g^{-1}$ body wt i.v. via the tail vein. Ro 03-8799 was injected over 35–40 sec to avoid vascular shock (Williams *et al.*, 1982). Ro 31-0313 was administered as an i.v. bolus. $^{51}Cr$ EDTA was diluted in HBSS to a concentration of 40 $\mu Ci\,ml^{-1}$ and injected i.v. as a bolus at either 0.005 or 0.01 $ml\,g^{-1}$ body wt via the tail vein. All substances were given 10 min before WBH, to allow peak tumour concentrations to be obtained.

*Hyperthermia*

The method of inducing WBH in mice was similar to that previously described by Honess and Bleehen (1982). Briefly, unanaesthetised unrestrained mice were enclosed in a wire mesh cage and placed under a fan in an incubator (type C2, Laboratory and Electrical Engineering Co., Nottingham, UK; approximate volume 3.5 $m^3$) set at 44°C. Fresh air was pumped continually into the incubator. Three to six mice were treated per 50 min session.

Temperatures were measured using a BAT-12 digital thermometer (±0.1°C) fitted with RET-3 murine rectal probes (Bailey's Instruments, Saddle Brooke, NJ, USA) or fine copper/constantan thermocouples as appropriate.

*Sample preparation*

Whole blood was removed under diethyl-ether anaesthesia by cardiac puncture into heparinised syringes. Plasma was obtained by centrifugation in a refrigerated Sorvall RC-5B Superspeed centrifuge (Du Point Instruments, USA) at 4000 g for 15 min. Nitroimidazoles were extracted from plasma by the addition of 10 vol acetonitrile (Walkerburn, Scotland, UK) containing internal standard (Ro 07-1902 at 2.5 mg $l^{-1}$) and centrifuged at 2000 g for 15 min. Aliquots of supernantant were evaporated to dryness *in vacuo* using a Savant Speed Vac Concentrator coupled to a Model 100A Refrigerated Condensation trap (Savant, Farmingdale, NY, USA). Residues were resuspended in running buffer prior to high-performance liquid chromatography (HPLC) analysis. After exsanguination animals were killed by cervical dislocation and tumour and brain samples rapidly excised and snap-frozen in dry ice at −70°C to prevent *ex vivo* metabolism. Individual tissue samples were homogenised (33% w/v in distilled water) in all-glass homogenisers before extraction and HPLC analysis as described for plasma. Samples were handled at 4°C and stored at −20°C for up to 4 weeks before analysis.

*High-performance liquid chromatography*

Concentrations of Ro 03-8799 and Ro 31-0313 in plasma and tissue homogenates were determined using the paired-ion, reverse-phase HPLC method of Malcolm *et al.* (1983), with minor modifications. Briefly, analyses were carried out using Waters modular HPLC equipment (Waters Assoc., Milford, Mass., USA) which included a Model 6000A chromatography pump, a Waters Intelligent Sample Processor (WISP), a model 440 fixed-wavelength UV detector and a Z-module. Separations were performed on Waters reverse-phase octadecylsilane (C18) Rad-Pak μBondapak columns (10 cm × 8 mm i.d., 10 μm beads) and eluted isocratically with 17% acetonitrile in 0.2 M glycine/hydrochloric acid buffer containing 5 mM heptane sulphonic acid (Fisons, Loughborough, UK), pH 2.45, at a flow rate of 4.5 ml $min^{-1}$. The absorbance of the effluent was monitored at 313 nm. Ro 03-8799 and Ro 31-0313 were identified by co-chromatography with authentic material and quantitated by peak-height ratio with reference to standard curves which were linear over the range 0.05–500 μg $ml^{-1}$. Same-day coefficients of variation for plasma spiked at 20 μg $ml^{-1}$ were 6.8% for Ro 03-8799 and 5.9% for Ro 31-0313 ($n=8$). The lower limit of detection was 0.05 μg $ml^{-1}$ giving an on-column detection of 1–2 ng for a 20 μl injection volume. Recoveries were always >95%. Run times were <6 min.

*$^{51}Cr$ EDTA clearance*

The effect of WBH on glomerular filtration rate (GFR) was assayed by measuring $^{51}Cr$ EDTA clearance (Chantler *et al.*, 1969). Aliquots of plasma from $^{51}Cr$ EDTA treated mice were counted for 5 min in a 1185 Series Automatic Gamma Counting System, Model 8931 (Searle Analytic Inc., Illinois, USA). Diluted injection solution was counted at the same time.

*Pharmacokinetic parameters*

Pharmacokinetic parameters were calculated as described in detail elsewhere (White & Workman, 1980; Workman & Brown, 1981) using a one- or two-compartment model with curve stripping as appropriate. Apparent volumes of distribution ($Vd_{ext}$ and $Vd_{area}$) were calculated using established methods (Wagner, 1975). Lines of best fit, with standard errors, were calculated by least-squares linear regression analysis yielding half-lives with 95% confidence limits. Plasma area under the concentration × time curve (AUC) was calculated from the expression $AUC_{0-\infty}=C_0/k$, where $C_0$ is the concentration at time 0 and $k$ is the elimination rate constant, or from the equation $AUC_{0-\infty}=A/\alpha+B/\beta$, as appropriate. Tissue $AUC_{0-t}$ from time 0 to time $t$ was estimated by Simpson's rule. The remaining $AUC_{t-\infty}$ was derived from $C_t/k$ for the tissue concerned.

*Determination of acute $LD_{50/7d}$*

The effect of WBH on the acute $LD_{50/7d}$ of Ro 03-8799 was determined using both tumour and non-tumour bearing mice, there being no apparent difference in response. Graded doses were given, ranging from 88 to 963 μg $g^{-1}$ using 3–5 mice per dose and 2–6 doses per experiment. Mice were observed for 7 days after treatment. $LD_{50/7d}$ values and confidence limits were derived using pooled data from 2 independent experiments by probit analysis, using the Generalised Interactive Modelling Programme (GLIM) of the Royal Statistical Society of London.

*Statistics*

Significance levels were determined by using Student *t*-test.

### Results

*Effects of WBH and Ro 03-8799 on core and tumour temperature*

Figure 1 shows the core (rectal) and central tumour temperature of unanaesthetised mice, lightly restrained with string or in perspex jigs, which were subjected to 50 min WBH in an incubator set at an air temperature of 44°C. Mice restrained in this way exhibited average core temperatures of 35°C prior to heating. Rectal temperatures increased steadily during the first 15 min of WBH, reaching a stable 41° ± 0.5°C for the rest of the heating period. Tumour core temperatures were 4–5°C below rectal values at the start of WBH, but rapidly equilibrated with rectal temperatures after the initial 10 min heating. Tumour cooling was also more rapid after WBH as compared to core temperatures. This heat treatment produced an average weight loss of 2.0, 3.9 and 6.4% after 10, 30 and 50 min WBH respectively.

Since some nitroimidazoles cause a decrease in body temperature (Gomer & Johnson, 1979; Workman, 1980) we determined the effects of 175 and 437 μg $g^{-1}$ Ro 03-8799 on rectal temperature in our mice. The results (Figure 2) show that the high dose produced a rapid drop of 2–3°C, with recovery to control temperatures after 2 h. Both the low dose and the vehicle control produced a slight transient drop of 0.5 and 1°C respectively, with recovery after ~1 h. Small numbers of mice were also administered HBSS vehicle, or Ro 03-8799 (175 or 437 μg $g^{-1}$) i.v. and subjected to WBH as described above. These animals showed similar temperature profiles to those in Figure 1.

*Effects of WBH on acute toxicity*

WBH markedly increased the acute toxicity of Ro 03-8799. Using pooled data from 2 independent experiments the $LD_{50/7d}$ was decreased 3-fold from 779 (725–836) to 259

**Figure 1** Effects of 50 min WBH (see **Material and Methods**) on rectal and KHT leg tumour temperature in unanaesthetised, lightly restrained C3H/He mice. Symbols: ■ incubator air temperature; ○ rectal temperature; and ● temperature at tumour centre. Results are mean (±2 s.e.) of 6 mice, from 5 independent determinations. In this and subsequent diagrams the shaded area indicates the time and duration of WBH after drug administration, and the sloped portion the initial thermal equilibration phase.

(217–310) μg g$^{-1}$ (95% confidence limits; $P<0.001$). Deaths in unheated animals occurred immediately after injection and usually during or shortly after WBH in heated animals.

Subsequent pharmacokinetic experiments were carried out at 175 μg g$^{-1}$ (~0.6 WBH $LD_{50/7d}$) with and without WBH, and at the higher, equitoxic dose of 437 μg g$^{-1}$ (~0.6 control $LD_{50/7d}$ without heat. No deaths occurred at these doses.

**Figure 2** The effects of various doses of Ro 03-8799 on the rectal temperature of C3H/He mice. Symbols: ▽ HBSS vehicle control, ● 175 μg g$^{-1}$ Ro 03-8799 i.v.; ■ 437 μg g$^{-1}$ Ro 03-8799 i.v. and △ ambient temperature. Results are from a typical experiment showing the mean ± 2 s.e. with 6 mice per point.

*Effects of WBH on Ro 03-8799 plasma pharmacokinetics*

Figure 3A shows the plasma elimination time course for Ro 03-8799 after 175 μg g$^{-1}$ i.v. Plasma clearance was biphasic. The distribution (α) phase was rapid, the $t_{1/2}\alpha$ being ~2 min, and essentially complete before heating began. The terminal (β) phase was much slower, with a $t_{1/2}\beta$ (with 95% confidence limits) of 23.5 (21.8–25.4) min, in good agreement with previous values at this dose (Stratford *et al.*, 1982). Since in each of two experiments the exposure during the α-phase was small ($Vd_{ext}/Vd_{area}=8.8$ and 7.4% respectively) the elimination kinetics were treated as monoexponential and fitted to a one-compartment model (see Dvorchik and Vessel, 1978). Table I and Figure 3a show that WBH increased plasma drug concentrations and prolonged the $t_{1/2}$ by 26% ($P<0.01$). There was no alteration in the apparent volume of

**Figure 3** Effects of WBH on the pharmacokinetics of Ro 03-8799 in (a) plasma, (b) KHT tumour and (c) brain from C3H/He mice. Symbols: △ 437 μg g$^{-1}$ Ro 03-8799 i.v.; ○ 175 μg g$^{-1}$ Ro 03-8799 i.v.; and ● 175 μg g$^{-1}$ i.v. + WBH. Pooled data from 2–3 independent experiments with 3–7 mice per point. Results are mean ± 2 s.e.

distribution ($Vd_{ext}$) despite the decrease in total body weight. Plasma $AUC_{0-\infty}$ for heated mice was increased by 22%, and the plasma clearance ($P_{cl}$) correspondingly reduced. Although the increase in plasma concentrations by WBH was quite marked, these were still substantially less than those in unheated animals treated with the equitoxic high-dose of Ro 03-8799 (Figure 3a). Thus increased plasma drug exposure alone could not account for the WBH enhanced acute lethality.

*Effects of WBH on Ro 03-8799 tumour and brain pharmacokinetics*

The effects of WBH on tumour and brain concentrations of Ro 03-8799 and on various tissue pharmacokinetic parameters are summarised in Figure 3 (panel b and c) and Table I respectively. In contrast to the plasma results, WBH had very little effect on tissue drug concentrations. There was no significant difference in the elimination half-lives of Ro 03-8799 from tumour tissue in all three treatment groups ($P>0.05$). The tumour $AUC_{0-90\,min}$ for Ro 03-8799 in the WBH treated mice was very similar to control values, and only about a third of the $AUC_{0-90\,min}$ occurring in unheated mice treated with the equitoxic high dose. WBH did not significantly alter the brain tissue $t_{1/2}$ ($P>0.05$). By contrast, the unheated high-dose group had a significantly longer $t_{1/2}$ ($P<0.01$). WBH had no effect on the brain $AUC_{0-90\,min}$, and the value was still only 40% of that for normothermic high-dose brain.

Table II shows the tumour/plasma and brain/plasma ratios for heated and control mice administered 175 $\mu g\,g^{-1}$ Ro 03-8799 i.v. Tumour/plasma ratios exceeded 100% after 10 min in all animals, and were constant between 20–120 min. WBH consistently reduced these ratios but this decrease was significant only at 40 min ($P<0.05$). However, the average tumour/plasma ratio over the period 20–120 min was $224 \pm 34\%$ (2 s.e., $n=25$) for controls compared to $164 \pm 20\%$ ($n=31$) for WBH treated animals, and this difference was highly significant ($P<0.01$). Unheated mice administered

**Table I** A summary of the effects of WBH on the pharmacokinetics of Ro 03-8799 in plasma, KHT tumour and brain tissue from C3H/He mice

| Tissue | Parameter | Treatment: Control | Treatment: +WBH | Treatment: Unheated high dose |
|---|---|---|---|---|
| Plasma | Dose ($\mu g\,g^{-1}$) | 175 | 175 | 437 |
| | $t_{1/2}$ (min) | 23.5 (21.8–25.4) | 29.1[b] (27.5–31.0) | 28.2[a] (25.3–31.8) |
| | $P_{cl}$ ($ml\,g^{-1}\,h^{-1}$) | 3.89 | 3.19 | 3.77 |
| | $Vd_{ext}$ ($ml\,g^{-1}$) | 2.20 | 2.23 | 2.55 |
| | $AUC_{0-\infty}$ ($\mu g\,ml^{-1}\,h$) | 44.9 | 54.8 | 116 |
| Tumour | $t_{1/2}$ (min) | 28.1 (25.9–30.8) | 31.8 (27.8–37.0) | 28.6 (23.3–37.0) |
| | $AUC_{0-90\,min}$ ($\mu g\,g^{-1}\,h$) | 61.6 | 56.7 | 177 |
| Brain | $t_{1/2}$ (min) | 31.0 (27.3–35.8) | 33.9 (30.3–38.4) | 44.5[b] (38.2–53.3) |
| | $AUC_{0-90\,min}$ ($\mu g\,g^{-1}\,h$) | 85.5 | 89.3 | 222 |

Parameters were derived using pooled data from 2 independent experiments which were fitted to a one compartment model. Ninety-five percent confidence limits are shown in parentheses. Each experiment involved 4–8 time points with 3–7 mice per point.
[a]$P<0.05$ and [b]$P<0.01$ significantly different from control.

**Table II** Effects of WBH on tissue/plasma ratios for Ro 03-8799 (175 $\mu g\,g^{-1}$) in C3H/He mice

| | Time after drug administration (min) | Tumour/plasma (%) Control | Tumour/plasma (%) +WBH | Brain/plasma (%) Control | Brain/plasma (%) +WBH |
|---|---|---|---|---|---|
| | 10 | 123±32 ($n=10$) | | 138±20 ($n=10$) | |
| Heating period | 20 | 184±25 ($n=6$) | 175±28 ($n=6$) | 207±46 ($n=6$) | 220±32 ($n=6$) |
| Heating period | 40 | 203±12 ($n=3$) | 149±20[a] ($n=6$) | 315±30 ($n=3$) | 249±56 ($n=6$) |
| Heating period | 60 | 250±96 ($n=6$) | 139±28 ($n=6$) | 432±62 ($n=6$) | 281±54[b] ($n=6$) |
| | 90 | 209±52 ($n=5$) | 145±72 ($n=7$) | 433±136 ($n=5$) | 307±32 ($n=7$) |
| | 120 | 266±108 ($n=5$) | 213±20 ($n=6$) | 471±60 ($n=5$) | 364±54[a] (n=6) |

Results are mean ± 2 s.e., using pooled data from 2 independent experiments.
[a]$P<0.05$ and [b]$P<0.01$ significantly different from control.

high-dose Ro 03-8799 had a mean tumour/plasma ratio of 226% between 20–90 min, very similar to the low dose unheated group.

Interestingly, Ro 03-8799 concentrated in brain to a much greater extent than in tumour (Table II). In unheated low-dose treated animals equilibration occurred after 40 min, giving a mean steady-state brain/plasma ratio of 444% between 60–120 min. WBH also reduced brain/plasma ratios, e.g. from 433 to 307% at 90 min, and this decrease was significant at 60 and 120 min ($P<0.01$ and $P<0.05$ respectively). In addition, heated brain/plasma ratios did not reach equilibration and tended to increase over the whole time course. Brain/plasma ratios in unheated mice treated with high-dose Ro 03-8799 were very similar to those occurring in unheated low-dose treated animals, being for example 412 and 415% at 60 and 90 min respectively.

The above results show that the enhanced acute toxicity of Ro 03-8799 induced by WBH does not result from an increase in drug concentration over the whole brain.

*Effects of WBH on the concentrations of the Ro 03-8799 N-oxide metabolite, Ro 31-0313*

Figure 4 shows the effects of WBH on the plasma concentrations of Ro 31-0313, the *N*-oxide metabolite of Ro 03-8799 (Malcom *et al.*, 1983), following Ro 03-8799 administration. WBH greatly increased the plasma concentration of Ro 31-0313. The metabolite $AUC_{0-\infty}$ for the WBH treated mice increased by 57% from 65.6 to 103 $\mu g\,ml^{-1}\,h$, a value only 17% less than that for the high dose unheated mice. This suggested that Ro 31-0313 might be responsible for the enhanced toxicity of Ro 03-8799 with WBH. However, acute toxicity experiments showed that, when administered i.v., Ro 31-0313 was considerably less toxic than Ro 03-8799 ($LD_{50/7d} > 1000\,\mu g\,g^{-1}$).

KHT tumour Ro 31-0313 metabolite concentrations were unaltered by WBH and did not exceed 4 $\mu g\,g^{-1}$ in any treatment group. Tumour/plasma ratios were 38–69% at 60–90 min.

Concentrations of the *N*-oxide in brain tissue were consistently just at or below the limit of detection ($\sim 1.0\,\mu g\,g^{-1}$ Ro 31-0313) and no increase was seen with WBH.

*The effects of WBH on the pharmacokinetics of i.v. administered Ro 31-0313*

To assess whether increased metabolism or decreased clearance were responsible for the elevated plasma *N*-oxide concentrations seen after 175 $\mu g\,g^{-1}$ Ro 03-8799 and WBH, we determined the effects of WBH on the plasma pharmaco-

**Figure 4** Effects of WBH on the concentrations of the N-oxide metabolite, Ro 31-0313, in C3H/He mice after Ro 03-8799 administration. Symbols: plasma Ro 31-0313 concentrations after, ◇ 175 $\mu g\,g^{-1}$ Ro 03-8799 i.v.; ◆ 175 $\mu g\,g^{-1}$ Ro 03-8799 plus WBH; and □ 436 $\mu g\,g^{-1}$ Ro 03-8799 i.v. Pooled data from 2 independent experiments with 3–7 mice per point. Results are mean ± 2 s.e.

**Figure 5** Plasma concentrations of the N-oxide metabolite Ro 31-0313 in C3H/He mice after 40 $\mu g\,g^{-1}$ Ro 31-0313 i.v. Symbols: ● with WBH; and ○ without. Data from 3 independent experiments with 3–8 mice per point. Results are mean ± 2 s.e.

kinetics of i.v. administered Ro 31-0313 (40 $\mu g\,g^{-1}$). This dose gave *N*-oxide concentrations similar to those occurring as a metabolite after 175 $\mu g\,g^{-1}$ Ro 03-8799. The results are summarised in Figure 5 and table III.

After i.v. administration Ro 31-0313 clearance was biphasic, and the data required analysis by the two-

**Table III** Effects of WBH on the pharmacokinetics of i.v. administered Ro 31-0313 in C3H/He mice

| *Parameters* | *Control* | *+ WBH* |
|---|---|---|
| Dose ($\mu g\,g^{-1}$) | 40 | 40 |
| A ($\mu g\,ml^{-1}$) | 101 | 101 |
| | (62.2–162) | (53.4 – 173) |
| B ($\mu g\,ml^{-1}$) | 15.2 | 22.2 |
| | (11.3–20.4) | (13.9–35.5) |
| $t_{1/2}\alpha$ (min) | 3.62 | 2.99 |
| | (3.04–4.49) | (2.50–3.73) |
| $t_{1/2}\beta$ (min) | 23.1 | 21.6 |
| | (20.5–26.6) | (17.9–27.1) |
| $AUC_{0-\infty}$ ($\mu g\,ml^{-1}\,h$) | 17.2 | 18.5 |
| $P_{cl}$ ($ml\,g^{-1}\,h^{-1}$) | 2.32 | 2.17 |
| $Vd_{area}$ ($ml\,g^{-1}$) | 1.29 | 1.13 |

Parameters were derived using pooled data from 3 independent experiments which were fitted to the 2 compartment open model:
$C_t = Ae^{-\alpha t} + Be^{-\beta t}$. Each experiment involved 5–7 time points with 3–8 mice per point.

compartment open model. In normothermic mice the α-phase $t_{1/2}$ was 3.62 (3.03–4.44) min while the β-phase $t_{1/2}$ was 23.1 (20.5–26.6) min. Plasma Ro 31-0313 concentrations were consistently higher in WBH treated animals compared to controls and this difference was clearly significant at 20 min ($P < 0.01$) but not at later times ($P > 0.05$). Although the change in total AUC was modest, the contribution from the β-phase was in fact increased by 36%, In addition, the apparent volume of distribution ($Vd_{area}$) was reduced by 13%.

### *Effects of WBH on the N-oxidation of Ro 03-8799 in mice*

Table IV compares the effects of WBH on the plasma concentrations of Ro 31-0313, either produced as a metabolite of Ro 03-8799 or administered i.v. After the first 40 min there is a significantly ($P < 0.01$) greater elevation in *N*-oxide concentration in mice treated with Ro 03-8799 and WBH compared to those treated with Ro 31-0313 and WBH. This shows that in addition to its effects on Ro 31-0313 clearance described above, WBH also enhances its metabolic production from Ro 03-8799.

### *The effects of WBH on $^{51}Cr$ EDTA clearance*

Since Ro 03-8799 is eliminated by both metabolic and renal clearance (Walton *et al.*, 1985) we investigated the effects of WBH on GFR using $^{51}Cr$ EDTA (Chantler *et al.*, 1969). Figure 6 shows that WBH almost completely inhibits $^{51}Cr$ EDTA clearance specifically during the heating period, resulting in a two-fold decrease in total plasma clearance from 1.14 to 0.58 $ml\,g^{-1}\,h^{-1}$.

**Table IV** Effect of WBH on plasma Ro 31-0313 concentrations in WBH treated C3H/He mice given either Ro 03-8799 (175 $\mu g\,g^{-1}$) or Ro 31-0313 (40 $\mu g\,g^{-1}$) i.v.

| *Time after injection (min)* | *Percentage increase in plasma Ro 31-0313 concentrations in WBH treated mice compared to unheated controls* | | | |
|---|---|---|---|---|
| | After Ro 03-8799 | Mean (±2 s.e.) | After Ro 31-0313 | Mean (±2 s.e.) |
| 20 | 17 | | 34 | |
| 40 | 63 | | 29 | |
| 60 | 81 | 74.5[a] | 60 | 36.8[a] |
| 90 | 80 | (8.3) | 24 | (16.0) |
| 120 | 74 | | 34 | |

These values were derived using the data shown in **Figures 4** and **5**.
[a]Significantly different from each other ($P < 0.01$).

**Figure 6** The effect of WBH on the plasma clearance of $^{51}Cr$ EDTA. Symbols: ○ unheated mice; and ● heated mice. Results are mean ± 2 s.e. with 3–8 mice per point. Data from 2 independent experiments.

### Discussion

In the present study we show that WBH markedly enhances the acute toxicity of the basic radiosensitizer Ro 03-8799 in mice. We also show that WBH has a complex effect on Ro 03-8799 pharmacokinetics. It significantly reduces the plasma clearance of parent drug but with no alteration in tumour and brain drug levels, resulting in reduced tissue/plasma ratios. WBH also increases plasma levels of the major metabolite of Ro 03-8799 by stimulating its production and decreasing its clearance through a reduction in volume of distribution.

Despite increasing interest in drugs combined with hyperthermia there have been very few studies of its effects on drug pharmacokinetics and metabolism. Mimnaugh *et al.* (1978) showed that WBH (42.3°C × 60 min) did not alter adriamycin $t_{1/2}\beta$ in rabbits, but slighly decreased $t_{1/2}\alpha$. In dogs WBH (43°C × 60 min) had no effect on plasma adriamycin fluorescence (Daly *et al.*, 1984). By contrast, Honess *et al.* (1985) found the plasma clearance of melphalan was reduced by 20–50% with WBH (41°C × 40 min) in mice; this was attributed mainly to a change in the apparent volume of distribution rather than elimination $t_{1/2}$. Using almost identical heating conditions to the latter study, we show here that Ro 03-8799 clearance was reduced by 18% and elimination $t_{1/2}$ increased by 26%, resulting in higher plasma drug concentrations; there was however no change in distribution volume. WBH also altered the pharmacokinetics of i.v. administered Ro 31-0313, the *N*-oxide metabolite of Ro 03-8799. Total plasma *N*-oxide concentrations were increased with hyperthermia, particularly during the β-phase. This was accompanied by a 6% decrease in plasma clearance, but no marked alteration in $t_{1/2}\alpha$ or $t_{1/2}\beta$. The total AUC was only slightly increased despite a 36% increase in the β-phase component. The 6% loss of body weight associated with this heating technique may also have contributed to these pharmacokinetic changes.

In view of the number of processes involved in drug disposition *in vivo*, it is perhaps not surprising that the pharmacokinetic effects of WBH varies between drugs. Further comparative studies are required to see if some general principles can be established.

Ro 03-8799 is a basic drug designed to concentrate in acid urine in the hope of accelerating urinary excretion and thereby reducing body exposure (Wardman, 1979). Ro 03-8799 is eliminated rapidly not only by urinary excretion but also by metabolic *N*-oxidation in both mice (Walton *et al.*, 1985) and man (Roberts *et al.*, 1986; Allen *et al.*, 1984). The precise mechanism of urinary elimination of Ro 03-8799, in particular the relative importance of active secretion and reabsorption versus simple glomerular filtration, is unknown. We show here that glomerular filtration is inhibited almost completely during the heating period, returning to normal immediately afterwards. This will contribute to reduced Ro 03-8799 clearance but cannot be the complete explanation as drug elimination was decreased uniformly over the whole time course and by only 18% compared to the 49% reduction in GFR. Similar reductions (51%) occurred in humans subjected to combined dehydration, exercise and mild heat stress (Smith *et al.*, 1952), an effect mainly attributed to decreased renal blood flow (Radigan & Robinson, 1950). Reduction in plasma pH would also decrease renal clearance by reducing the pH gradient between plasma and urine, and hence the rate of Ro 03-8799 accumulation into acid urine. Such a change may occur in mice during WBH due to metabolic acidosis, and blood pH is markedly decreased in dogs after WBH (Macy *et al.*, 1985).

A novel feature of this basic radiosensitizer is its ability to concentrate in tissue giving tumour/plasma and brain/plasma ratios of 200%–400% after equilibration (Allen *et al.*, 1984; Roberts *et al.*, 1986). In unheated mice, high-dose Ro 03-8799 (437 $\mu g g^{-1}$) produced higher plasma and tissue concentrations compared to low doses (175 $\mu g g^{-1}$) although tissue/plasma ratios were very similar. By contrast WBH increased low-dose Ro 03-8799 plasma concentrations without affecting those in tumour and brain, resulting in reduced tissue/plasma ratios. This failure of heated tissue to equilibrate with the increased plasma drug concentrations after WBH may involve a complex interaction of several factors such as drug delivery and uptake, tissue pH and/or drug metabolism.

A reduction in blood perfusion would impair tissue uptake leading to a decreased tissue/plasma ratio. However, tissue blood flow tends to increase at temperatures up to 42°C, with vascular stasis occurring predominantly at higher temperatures and longer times than those used here (see Reinhold *et al.*, 1985).

WBH may also decrease tissue pH through stimulation of anaerobic glycolysis (see Reinhold *et al.*, 1985). Our measurements do not distinguish intracellular from extracellular drug and we have no data on intracellular/extracellular pH changes after WBH. In view of these unknown variables it is difficult to predict the effects of possible pH changes on overall tissue concentrations. Simplistically, however, a reduction in tissue pH would be likely to increase the total amount of the basic drug by ion-trapping, leading to a higher tissue/plasma ratio.

Little is known about the effects of hyperthermia on drug metabolism. The reduced Ro 03-8799 tissue/plasma ratios after WBH may reflect heat-stimulated reductive metabolism to non-UV absorbing metabolites, (Schwartz & Hofheinz, 1982). The possibility that such differences resulted from *ex vivo* metabolism were minimised by rigorous sample handling techniques. Honess *et al.* (1980) reported 50–70% lower tumour MISO concentrations in locally heated mouse leg tumours; but this may have occurred through reduced drug uptake as well as enhanced reductive metabolism. Other workers demonstrated increased levels of reduced adriamycin metabolities, particularly aglycones, in locally heated mouse tumours (Magin *et al.*, 1980) as well as other normal rabbit tissues following WBH (Mimnaugh *et al.*, 1978). In more direct support of our hypothesis we have recently shown that mild hyperthermia (41°C) can enhance benznidazole nitro-reduction *in vitro* (Walton *et al.*, unpublished results).

We also present evidence of hyperthermia-potentiated oxidative metabolism of Ro 03-8799 to its *N*-oxide, Ro 31-0313. *N*-oxidation is regarded as predominantly involving components of the hepatic mixed-function oxidases (MFO) (Bickel, 1969). We have also shown that hyperthermia (41°C) enhances oxidative O-demethylation of MISO by MFO *in vitro* (Walton, Bleehen & Workman, unpublished results). In contrast Collins and Skibba (1983), showed using an isolated rat liver perfusion technique, that the $t_{1/2}$s of antipyrine and cyclophosphamide were increased at 42 compared to 37°C, concluding that hyperthermia depressed hepatic MFO activity. This may reflect the different model systems and heat doses employed, as well as the particular drugs and metabolic reactions involved.

The 3-fold increase in Ro 03-8799 acute lethality with WBH is similar to the doubling of MISO acute toxicity by local hyperthermia, where core temperatures approached 41°C (Overgaard, 1979; 1980). The nature of Ro 03-8799 acute toxicity, resulting in death immediately after administration in normothermic mice and during or shortly after WBH in heated mice, is at present unknown. At lethal doses of Ro 03-8799 normothermic mice suffered rapid convulsions characteristic of central nervous system toxicity. This may be related to the acute CNS affect seen with Ro 03-8799 in man (Roberts *et al.*, 1986; Saunders *et al.*, 1984). Considerable evidence suggests that exposure of the nervous system to nitroimidazoles is correlated to their neurotoxicity (e.g. Brown & Workman, 1980; Conroy *et al.*, 1982). However, we found brain Ro 03-8799 concentrations were minimally altered by WBH, being substantially less than in unheated animals treated with equitoxic high doses. It seems that the increased lethality cannot be attributed to elevated brain exposure to the parent drug. However, reductive metabolism of nitroimidazoles generates highly reactive and toxic metabolities (Rauth, 1984). Consequently increased toxicity may result from hyperthermia-enhanced Ro 03-8799 reductive metabolism.

Other organ toxicities also occur, e.g., chronic Ro 03-8799 administration caused severe hepatotoxicity in monkeys (Roche Products, 1982). WBH tends to produce liver temperatures 1–2°C above core temperatures, also resulting in hepatotoxicity after prolonged exposures (Fletcher *et al.*, 1982). Thus liver damage may be important with the combination. If different organ toxicities do operate with Ro 03-8799 in unheated and heated animals, correlation with the measured pharmacokinetic parameters would not be expected.

High nitroimidazole doses produce large decreases in mouse core temperature (e.g. Workman, 1980) and this has a protective effect on the acute lethality of MISO (Gomer & Johnson, 1979) possibly through decreased metabolic activity. Thus the enhanced toxicity of high doses of Ro 03-8799 with WBH may arise from abolition of the protective hypothermia together with an attendant increase in Ro 03-8799 reductive activation.

In conclusion, WBH substantially increased the acute toxicity of Ro 03-8799. It also increased plasma Ro 03-8799 concentrations, partly as a result of inhibited glomerular filtration. Absolute tumour and brain drug concentrations were unaltered, giving reduced tissue/plasma ratios. These WBH-induced alterations in Ro 03-8799 pharmacokinetics probably contribute to its enhanced toxicity but cannot completely explain it. There was no clear correlation between brain drug levels and increased toxicity. However, WBH did enhance the hepatic *N*-oxidation of Ro 03-8799 *in vivo*, and hyperthermia potentiated metabolism, particularly nitro-reduction, may be involved in the increased toxicity of this combination. In view of this possibility and the cytotoxic potential of bioreductive metabolism, we are investigating the value of Ro 03-8799 or other bioreductively activated drugs in combination with localised tumour hyperthermia.

We thank Dr Carey Smithen (Roche, Welwyn Garden City, UK) for supplies of the nitroimidazoles. We are grateful to J. Shaw for care of the animals and Fiona Brown and Pearl Glasscock for typing the manuscript. M. Walton also thanks the Cancer Research Campaign for a research studentship award.

### References

ADAMS, G.E., FLOCKHART, I.R., SMITHEN, C.E., STRATFORD, I.J., WARDMAN, P. & WATTS, M.E. (1976). Electron-affinic sensitization. VII: A correlation between structures, one-electron reduction potential and efficiencies of nitroimidazoles as hypoxic radiosensitizers. *Radiat. Res.*, **67**, 9.

ALLEN, J.G., DISCHE, S., LENOX-SMITH, I., MALCOLM, S.L. & SAUNDERS, M.I. (1984). The pharmacokinetics of a new radiosensitizer Ro 03-8799 in humans. *Eur. J. Clin. Pharmacol.*, **27**, 483.

BICKEL, M.H. (1969). The pharmacology and biochemistry of N-oxides. *Pharmac. Rev.*, **21**, 325.

BLEEHEN, N.M., HONESS, D.J. & MORGAN, J.E. (1977). Interaction of hyperthermia and the hypoxic-cell sensitizer Ro 07-0582 on the EMT6 mouse tumour. *Br. J. Cancer*, **35**, 299.

BROWN, J.M. & WORKMAN, P. (1980). Partition coefficient as a guide to the development of radiosensitizers which are less toxic that misonidazole. *Radiat. Res.*, **82**, 171.

CHANTLER, C., GARNETT, E.S., PARSONS, V. & VEALL, N. (1969). Glomerular filtration rate measurement in man by the single injection method using $^{51}$Cr EDTA. *Clin. Sci.*, **37**, 169.

COLLINS, F.G. and SKIBBA, J.L. (1983). Altered hepatic functions and microsomal activity in perfused rat liver by hyperthermia combined with alkylating agents. *Cancer Biochem. Biophys.*, **6**, 205.

CONROY, P.J., MCNEILL, T.H., PASSALACQUA, W., MERRITT, J., REICH, K.R. & WALKER, S. (1982). Nitroimidazole toxicity: Are mouse studies predictive. *Int. J. Radiat. Biol. Phys.*, **8**, 799.

DALY, J.M., WANG, Y.M., KAPELANSKI, D. & HOWARD-FRAZIER, O. (1984). Systemic thermochemotherapy, toxicity and plasma pharmacokinetics of methotrexate and doxorubicin. *J. Surg. Res.*, **37**, 343.

DENNIS, M.F., STRATFORD, M.R.L., WARDMAN, P. & WATTS, M.E. (1985). Cellular uptake of misonidazole analogues with acidic or basic functions. *Int. J. Radiat. Biol.*, **47**, 629.

DVORCHIK, B.H. and VESELL, E.S. (1978). Significance of error associated with use of the one-compartment formula to calculate clearance of thirty-eight drugs. *Clin. Pharmacol. Ther.*, **23**, 617.

FLETCHER, A.M., CETAS, T.C., DEWHIRST, M.W. & WILSON, S.E. (1982). Liver temperatures in rats subjected to whole-body hyperthermia: Evidence for congestion and lymphocytic damage. *Natl Cancer Inst. Monogr.*, **61**, 231.

GEORGE, K.C., HIRST, D.G. & MCNALLY, N.J. (1977). Effect of hyperthermia on the cytotoxicity of the radiosensitizer Ro 07-0582 in a solid mouse tumour. *Br. J. Cancer*, **35**, 372.

GOMER, C.J. & JOHNSON, R.J. (1979). Relationship between misonidazole toxicity and core temperature in C3H mice. *Radiat. Res.*, **78**, 329.

HONESS, D.J., WORKMAN, P., MORGAN, J.E. & BLEEHEN, N.M. (1980). Effects of local hyperthermia on the pharmacokinetics of misonidazole in the anaesthetized mouse. *Br. J. Cancer*, **41**, 529.

HONESS, D.J. & BLEEHEN, N.M. (1982). Sensitivity of normal mouse marrow and RIF-1 tumour to hyperthermia combined with cyclophosphamide or BCNU: A lack of therapeutic gain. *Br. J. Cancer*, **46**, 236.

HONESS, D.J., DONALDSON, J., WORKMAN, P. & BLEEHEN, N.M. (1985). The effects of systemic hyperthermia on melphalan pharmacokinetics in mice. *Br. J. Cancer*, **51**, 77.

MACY, D.W., MACY, C.A., SCOTT, R.J., GILLETTE, E.L. & SPEER, J.F. (1985). Physiological studies of whole-body hyperthermia in dogs. *Cancer Res.*, **45**, 2769.

MAGIN, R.L., CYSYK, R.L. & LITTERST, K.L. (1980). Distribution of adriamycin in mice under conditions of local hyperthermia which improve systemic drug therapy. *Cancer Treat. Rep.*, **64**, 203.

MALCOLM, S.L., LEE, A. & GROVES, J.K. (1983). High-performance liquid chromatographic analysis of the new hypoxic-cell radiosensitizer Ro 03-8799, in biological samples. *J. Chromatog.*, **273**, 327.

MIMNAUGH, E.G., WARING, R.W., SIKIC, B.I. & 5 others (1978). Effects of WBH on the disposition and metabolism of adriamycin in rabbits. *Cancer Res.*, **38**, 1420.

OVERGAARD, J. (1979). Effects of local hyperthermia on the acute toxicity of misonidazole in mice. *Br. J. Cancer*, **39**, 96.

OVERGAARD, J. (1980). Effects of misonidazole and hyperthermia on the radiosensitivity of a C3H mouse mammary carcinoma and its surrounding normal tissue. *Br. J. Cancer*, **41**, 10.

RADIGAN, L.R. & ROBINSON, S. (1950). Effects of environmental heat stress and exercise on renal blood flow and filtration rate. *J. Appl. Physiol.*, **2**, 185.

RAUTH, A.M. (1984). Pharmacology and toxicology of sensitizers, mechanism studies. *Int. J. Radiat. Oncol. Biol. Phys.*, **10**, 1293.

REINHOLD, H.S., WIKE-HOOLEY, J.L. VAN DEN BERG, A. & VAN DEN BERG-BLOK, A. (1985). Environmental factors, blood flow and microcirculation. In *Hyperthermic Oncology*, Overgaard, J. (ed) p. 41. Taylor and Francis: London.

ROBERTS, J.T., BLEEHEN, N.M., WALTON, M.I. & WORKMAN, P. (1986). A clinical Phase I toxicity study of Ro 03-8799: Plasma, urine, tumour and normal brain pharmacokinetics. *Br. J. Radiol.*, **59**, 107.

SAUNDERS, M.I., ANDERSEN, P.J., BENNETT, M.H. & 4 others (1984). The clinical testing of Ro 03-8799 pharmacokinetics, toxicity, tissue and tumour concentrations. *Int. J. Radiat. Oncol. Biol. Phys.*, **10**, 1759.

SCHWARTZ, D.E. & HOFHEINZ, W. (1982). Metabolism of nitroimidazoles. In *Nitroimidazoles, Chemistry, Pharmacology and Clinical Applications*, Breccia, A., *et al.* (eds) p. 189. NATO Advanced Study Institute Series, Series A, *Life Sci.*, **42**, Plenum Press: New York.

SMITH, J.M., ROBINSON, S. & PEARCY, M. (1952). Renal responses to exercise, heat and dehydration. *J. Appl. Physiol.*, **4**, 659.

SMITHEN, C.E., CLARKE, E.D., DALE, J.A., JACOBS, R.S., WARDMAN, P., WATTS, M.E. & WOODCOCK, M. (1980). Novel (Nitro-1-imidazolyl) alkanolamines as potential radiosensitizers with improved therapeutic properties. In *Radiation Sensitizers*, Brady, L.W. (ed) p. 22. Masson: New York.

STRATFORD, I.J. & ADAMS, G.E. (1977). Effects of hyperthermia on differential cytotoxicity of a hypoxic-cell radiosensitizer Ro 07-0582, on mammalian cells *in vitro*. *Br. J. Cancer*, **35**, 307.

STRATFORD, M.R.L., MINCHINTON, A.L., STEWART, F.A. & RHANDAWA, V.S. (1982). Pharmacokinetic studies on some novel (2-nitro-1-imidazolyl) propanolamine radiosensitizers. In *Nitroimidazoles, Chemistry, Pharmacology and Clinical Applications* Breccia, A., *et al.* (eds) p. 165. NATO Advanced Study Institute Series, Series A, *Life Sci.*, **42**, Plenum Press: New York.

TWENTYMAN, P.R., KALLMAN, R.F. & BROWN, J.M. (1979). The effect of time between X-irradiation and chemotherapy on the growth of three solid mouse tumours–1. Adriamycin. *Int. J. Radiat. Oncol. Biol. Phys.*, **5**, 1255.

WALTON, M.I., BLEEHEN, N.M. & WORKMAN, P. (1985). The reversible N-oxidation of the radiosensitizer Ro 03-8799. *Biochem. Pharmacol.*, **34**, 3939.

WAGNER, J.G. (1975). In *Fundamentals of Clinical Pharmacokinetics* (1st edition) Drug Intelligence Publications Inc., Hamilton, Illinois.

WARDMAN, P. (1979). The chemical basis for the development of hypoxic-cell radiosensitizers. In *Radiosensitizers of hypoxic cells*, Breccia, A. *et al.* (eds) p. 91. Elsevier: Amsterdam.

WATTS, M.E. & JONES, N.R. (1985). The effects of extracellular pH on radiosensitization by misonidazole and acidic and basic analogues. *Int. J. Radiat. Biol.*, **47**, 645.

WHITE, R.A.S. & WORKMAN, P. (1980). Pharmacokinetic and tumour penetration properties of the hypoxic-cell radiosensitizer desmethylmisonidazole (Ro 05-9963) in dogs. *Br. J. Cancer*, **41**, 268.

WILLIAMS, M.V., DENEKAMP, J., MINCHINTON, A.L. & STRATFORD, M.R.L. (1982). *In vivo* assessment of basic 2-nitro-imidazole radiosensitizers. *Br. J. Cancer*, **46**, 127.

WORKMAN, P. (1980). Dose-dependence and related studies on the pharmacokinetics of misonidazole and desmethylmisonidazole in mice. *Cancer Chemother. Pharmacol.*, **5**, 27.

WORKMAN, P. & BROWN, J.M. (1981). Structure-pharmacokinetic relationships for misonidazole analogues in mice. *Cancer Chemother. Pharmacol.*, **6**, 39.

WORKMAN, P. (1983). Pharmacokinetics of radiosensitizing agents. In *Pharmacokinetics of Anticancer Agents in Humans* Adams, G. *et al.* (eds) p. 291. Elsevier: Amsterdam.